# Characterization of a Surface Glycoprotein from *Echinococcus multilocularis* and Its Mucosal Vaccine Potential in Dogs

**DOI:** 10.1371/journal.pone.0069821

**Published:** 2013-07-23

**Authors:** Hirokazu Kouguchi, Jun Matsumoto, Ryo Nakao, Kimiaki Yamano, Yuzaburo Oku, Kinpei Yagi

**Affiliations:** 1 Department of Infectious Diseases, Hokkaido Institute of Public Health, Sapporo, Japan; 2 Laboratory of Medical Zoology, Nihon University College of Bioresource Sciences, Fujisawa, Kanagawa, Japan; 3 Division of Bioinformatics, Research Center for Zoonosis Control, Hokkaido University, Sapporo, Hokkaido, Japan; 4 Department of Parasitology, School of Veterinary Medicine, Faculty of Agriculture, Tottori University, Tottori, Japan; Federal University of São Paulo, Brazil

## Abstract

Alveolar echinococcosis is a refractory disease caused by the metacestode stage of *Echinococcus multilocularis*. The life cycle of this parasite is maintained primarily between foxes and many species of rodents; thus, dogs are thought to be a minor definitive host except in some endemic areas. However, dogs are highly susceptible to *E. multilocularis* infection. Because of the close contact between dogs and humans, infection of dogs with this parasite can be an important risk to human health. Therefore, new measures and tools to control and prevent parasite transmission required. Using 2-dimensional electrophoresis followed by western blot (2D-WB) analysis, a large glycoprotein component of protoscoleces was identified based on reactivity to intestinal IgA in dogs experimentally infected with *E. multilocularis*. This component, designated SRf1, was purified by gel filtration using a Superose 6 column. Glycosylation analysis and immunostaining revealed that SRf1 could be distinguished from Em2, a major mucin-type antigen of *E. multilocularis*. Dogs (n = 6) were immunized intranasally with 500 µg of SRf1 with cholera toxin subunit B by using a spray syringe, and a booster was given orally using an enteric capsule containing 15 mg of the same antigen. As a result, dogs immunized with this antigen showed an 87.6% reduction in worm numbers compared to control dogs (n = 5) who received only PBS administration. A weak serum antibody response was observed in SRf1-immunized dogs, but there was no correlation between antibody response and worm number. We demonstrated for the first time that mucosal immunization using SRf1, a glycoprotein component newly isolated from *E. multilocularis* protoscoleces, induced a protection response to *E. multilocularis* infection in dogs. Thus, our data indicated that mucosal immunization using surface antigens will be an important tool to facilitate the development of practical vaccines for definitive hosts.

## Introduction

Alveolar echinococcosis (AE) is a serious public health problem in many endemic regions of the Northern Hemisphere [1]. Within the genus *Echinococcus*, the species that have major public health importance are *Echinococcus granulosus*, the causative agent of cystic echinococcosis (CE), and *E. multilocularis*, the causative agent of AE [2]. The life cycle of *E. multilocularis* is maintained between intermediate hosts, mainly rodents in the wild and definitive host wild carnivores. In human AE, infection is caused by accidental ingestion of parasite eggs excreted in the feces of carnivores such as foxes and dogs. As much as 10 or more years after the initial infection, metacestodes proliferate unrestrictedly into the liver and other organs, forming a tumor-like mass, which may cause organ dysfunction. Although the prevalence of *E. multilocularis* infection in humans is generally low, AE can be highly lethal because of the unlimited capacity for proliferation and metastasis of the parasitic lesions, unless appropriate treatment is administered.

Normally, the major definitive hosts of *E. multilocularis* are wild foxes, and dogs are not considered to play an important role in the natural transmission of the parasite, with the exception of highly endemic areas such as western China and part of Alaska [3]–[5]. However, dogs also possess high susceptibility to experimental infection with the adult parasite, suggesting that accidental infection of dogs with *E. multilocularis* could be an important source of AE infection in humans because of their close contact with their owners.

To control this zoonotic disease, some prevention programs have been implemented in various endemic areas. Distribution of baits containing praziquantel is an effective measure for reducing the infection rate of *E. multilocularis* in wild foxes. Some studies have reported that bait distribution achieved significant level of (from 30 to 50%) reduction in the prevalence of *E. multilocularis* within 18 months [5]–[8]. Likewise, prevention programs, including repeated treatment of dogs with praziquantel and health education for dog owners, resulted in a significant reduction in *E. granulosus* infections [9]. However, to maintain such effectiveness, it is necessary to conduct these prevention programs over a long-term period, which would place a significant economic burden on society. Therefore, it is necessary to establish new measures to reduce the risk of parasite transmission from the definitive host to humans. The development of effective vaccines can provide new measures for the long-term control of this parasite.

Limited knowledge is available regarding immunology-based protective responses to *Echinococcus* infection in definitive hosts due to controversial reports [10]. In fact, a few studies have demonstrated acquired immunity to *E. multilocularis* in canids. In particular, whether this parasite stimulates an acquired immune response in the intestines of canids is still debatable [11]. Tanaka et al. showed that repeated experimental infection in 2 dogs with *E. multilocularis* resulted in a significant reduction in worm burden in dogs [12]. Similar results were observed in dogs infected with *E. granulosus* [13]. Early attempts, including generating immunity in dogs through vaccination with various native antigens from *E. granulosus*, induced significant levels of protection [14]–[16]. Recently, Zhang et al. showed that subcutaneous vaccination with a series of *egM* recombinant antigens provided very high levels of protection against *E. granulosus* in dogs [17]. Petavy et al. reported that an oral recombinant vaccine against *E. granulosus* showed promise with respect to resisting CE in dogs [18]. These experimental results suggest that prevention of the disease by vaccination is possible and that dogs can generate a high degree of protective immunity against parasites. On the other hand, these above-mentioned reports have been criticized in terms of their statistical analyses [10]; therefore, additional supporting data are needed. Moreover, there has been little progress in the development of a vaccine against *E. multilocularis* in dogs.

IgA is widely accepted as a protective molecule in the gut; in particular, IgA binds to bacteria or gut-dwelling parasites, exerting its key function as an initial barrier to infection. Thus, research in mucosal immunology is focused on developing new approaches for mucosal vaccines [19].

In this report, we identified a potential vaccine candidate based on reactivity to intestinal IgA from dogs infected with *E. multilocularis*. We describe, for the first time, that this antigen shows mucosal vaccine potential against *E. multilocularis* infection in dogs.

## Materials and Methods

### Ethics Statement

This study was performed in strict accordance with the Guidelines for Animal Experimentation of the Japanese Association for Laboratory Animal Science, and the protocol for the animal experiments was approved by the ethics committee of the Hokkaido Institute of Public Health (permit number: K23-02). All the surgeries were performed under sodium pentobarbital anesthesia, and every effort was made to minimize suffering.

### Parasite Materials

*E. multilocularis* (Nemuro strain) was obtained from a dog-cotton rat life cycle routinely maintained at the Hokkaido Institute of Public Health. Protoscoleces were collected from cysts developed in cotton rats at 10–14 months after infection and were washed 7 times with 10 mM phosphate buffer (pH 7.4) containing 137 mM NaCl and 2.7 mM KCl (PBS) with penicillin G (500 IU/mL) and streptomycin (1 mg/mL). Adult worms were collected on day 35 postinfection from the infected dogs. The worms were first released from the intestinal contents by soaking them in PBS to remove intestinal mucus and were then rinsed several times in PBS. These materials were used to prepare vaccine antigens and crude extracts for 2D-WB analysis. All experiments in this study were performed in a specially designed safety facility (biosafety level 3) at the Hokkaido Institute of Public Health, Sapporo, Japan.

### Collection of Serum and Intestinal Swab Samples

All the dogs used in this study were purchased from Sankyo Lab Service Co. Ltd. (Sapporo, Japan). To collect serum and intestinal swab samples, 6 dogs (female beagles, 3–16 months old) were divided into 3 groups and were used for experimental infection. Dogs in group 1 were infected once. Dogs in groups 2 and 3 were infected 3 and 5 times, respectively. All experimental infections were performed by oral administration of 5×10^5^ *E. multilocularis* protoscoleces, and the infection was terminated on day 35 postinfection by administering praziquantel. Repeated infections were performed with at least 1-week intervals between infections.

Serum samples were collected every week after infection from infected dogs, and sera were stored individually at –30°C until use. Interstitial swab samples were collected from the small intestine of infected dogs. The small intestine was divided into 6 sections, and the central 3 sections of the intestine were opened with sterile scissors. Secretions were wiped as equivalently as possible with a sterile cotton swab and placed into tubes in 2 mL PBS containing protease inhibitor cocktail (Roche), streptomycin, and penicillin G. The tubes were immediately placed in liquid nitrogen and stored at –80°C until use. Each group of intestinal swab samples was pooled and used in 2D-WB analysis.

### Purification of a Vaccine Antigen from *E. multilocularis* Protoscoleces

Approximately 1.4 mL of the washed protoscoleces was transferred to a polystyrene test tube (17×100 mm) containing 5 mL PBS supplemented with 2.4% Triton X-100. Following gentle shaking for 3 min at room temperature, the suspension was sonicated 6 times for 10 s with a probe-type ultrasonic generator (UCD-130, Tosho Electronic Co.) at 4°C to obtain a crude extract of protoscoleces. The crude extract was centrifuged for 15 min at 3,500 rpm, and the clarified supernatant was dialyzed against distilled water overnight. The dialyzed solution was separated into aliquots of approximately 5 mL in a small vial and lyophilized. This sample was used as the protoscoleces crude extract (PCE). The PCE was dissolved in 1 mL distilled water and an appropriate amount of protease inhibitor cocktail (complete EDTA free, Roche). Two hundred microliters of the dissolved crude extract was centrifuged to remove insoluble materials and applied to a Superose 6 10/300GL column fitted on an AKTA explorer (GE Healthcare) equilibrated with PBS at a flow rate of 0.3 mL/min. The fraction eluted at the void volume (7.5 to 9 mL) was used as the vaccine antigen and was designated SRf1. Elution positions of thyroglobulin (669 kDa) and aldolase (158 kDa) were determined in a separate run under identical conditions. The purity of SRf1 was determined by densitometric analysis of Coomassie brilliant blue-stained SDS-PAGE gels by using the UN-SCAN-IT ver. 6.1 software (Silk Scientific, Inc., Orem, UT, USA). BSA was used as a standard.

### 2D-PAGE and Western Blotting

2D-PAGE followed by western blot (2D-WB) analysis was performed as described previously [20]. Briefly, 120 µg of crude extract from adult worms (i.e., ACE) or PCE was applied to 2D-PAGE. The separated proteins were stained with CBB or reagents in a Pro-Q Emerald 300 glycoprotein gel kit (Molecular Probes, Inc.), as recommended by the manufacturer. After 2D-PAGE, the separated proteins were electroblotted onto a PVDF membrane and blocked by incubating in blocking buffer (PBS containing 10% skim milk and 0.1% Tween 20) for 1 h. The membrane was incubated with the intestinal swab sample (1∶10 dilution in blocking buffer) or serum sample (1∶400 dilution in blocking buffer) for 1 h. After washing, the membrane was incubated with anti-dog IgA-alkaline phosphatase (AP) conjugate (1∶3000 dilution in blocking buffer) for detection of mucosal IgA or incubated with anti-dog IgG-AP conjugate (1∶3000 dilution in blocking buffer) for detection of serum IgG. The bound antibodies were detected with a BCIP/NBT immunodetection kit (PerkinElmer).

### Preparation of Specific Antiserum Against the SRf1 Antigen

Preparation of mouse anti-SRf1 antiserum was performed as described previously [21]. SRf1 was heat-treated in the presence of 1% SDS to increase immunogenicity. Approximately 50 µg of SRf1 protein was administered to balb/c mice with Freund’s complete adjuvant. Thereafter, 2 boosters with incomplete adjuvant were given to the animals at 2-week intervals.

### Enzyme-linked Immunosorbent Assay

Specific serum antibodies to SRf1 were measured by an indirect enzyme-linked immunosorbent assay (ELISA) as described previously [22]. A 96-well ELISA plate was coated with the antigen preparation for 5 h at 37°C. The antigens were diluted in 0.05 M carbonate buffer (pH 9.6) to a concentration of 10 µg/mL. The protein concentration was calculated by measuring the optical density at 280 nm with BSA as a standard (0.7 OD at 280 nm = 1 mg/mL). Each well was washed with PBS containing 0.05% Tween 20 (PBS/Tween). The wells were reacted with sample sera diluted 1∶100 with dilution buffer (1.0% [w/v] casein in PBS/Tween) overnight at 4°C. The wells were then washed with PBS/Tween and incubated with horseradish peroxidase-conjugated rabbit anti-dog IgG, IgA, or IgE at a dilution of 1∶3000 in dilution buffer for 1 h at 37°C. Following the final wash with PBS/Tween, substrate solution containing 0.04% *o*-phenylenediamine and 0.006% H_2_O_2_ in 100 mM citrate phosphate buffer (pH 5.0) was applied to each well. The plate was incubated at room temperature, and the optical density was read at 492 nm.

### Mucosal Immunization and Challenge Infection

A total of 17 dogs (female beagles, ages 3–4 months old) were used in this experiment. Six dogs were immunized nasally 4 times on days 0, 14, 28, and 42. Five hundred micrograms of SRf1 was mixed with 100 µg of cholera toxin (CT) subunit B (CTB, C9903, Sigma, St. Louis, MO, USA) in 400 µL PBS and incubated at 4°C overnight according to the method reported by Tuji et al. [23]. Before administration, carboxyvinyl polymer (CVP, SENKEN Co. LTD, Japan) was added to increase viscosity to a final concentration of 0.1%, and 0.1 µg of CT (Wako Pure Chemical Industries, Ltd.) was supplied to increase the immunogenicity of the antigen. The antigen (500 µg/animal) was administered to dogs nasally with a spray syringe (Nipro Corp., Japan). Thereafter, 3 boosters were given to each animal by oral administration of an enteric capsule (Sunsho Pharmaceutical Co. Ltd., Japan) containing 15 mg lyophilized SRf1 mixed with 100 µg CT on days 28, 42, and 56. Seven days after final administration of the capsule, all animals were orally administered 5×10^5^ *E. multilocularis* protoscoleces. On day 35 postinfection, animals were euthanized, and necropsies were performed. The small intestine was divided into 6 sections and incubated in DMEM at 4°C for 7 days. Naturally released and scraped worms were counted after appropriate dilutions. Two control groups received the same schedule of administration with PBS alone (n = 5) or adjuvant alone (n = 6) instead of SRf1.

### Evaluation of Protease Tolerance

Five hundred micrograms of SRf1 was digested in the presence of pepsin (1.0 mg/mL) or trypsin (0.4 mg/mL) and chymotrypsin (1.7 mg/mL) at 37°C for 1 and 4 h. The pH of the reaction mixture was adjusted to 2 for pepsin digestion or 7.4 for trypsin digestion. The reaction mixtures were analyzed by western blotting using sera from dogs infected 5 times and by gel filtration chromatography under identical conditions as mentioned above. The peak area was calculated with Unicorn ver. 3.10 equipped with an AKTA explorer system.

### Determination of O-glycosylation

SRf1 was lyophilized in a reaction vial, and anhydrous hydrazine was added to the vial. After replacement with nitrogen under reduced pressure, the sample was heated at 60°C for 6 h under reduced pressure. Hydrazine was removed by 3 rounds of toluene azeotrope, and the sample was reacted with saturated NaHCO_3_ and acetic anhydrate for 30 min. The reaction mixture was then applied to a Dowex AG50x2(H+) exchange resin and washed with distilled water 5 times. The eluate containing the released oligosaccharide was lyophilized.

Glycoblotting [24] and MALDI-TOF mass spectrometry (MS) analysis were performed according to the report described by Kato et al. [25], except for the use of 20 mM O-benzylhydroxylamine hydrochloric acid (BOA) for labeling. Purified BOA-labeled glycans were mixed with 2,5-dihydroxybenzoic acid solution and subsequently subjected to MALDI-TOF analysis by using an Autoflex III TOF/TOF mass spectrometer (Bruker Daltonics GmbH, Bremen, Germany). The compositions of glycan structures were estimated with GlycoMod software (http://www.expasy.org/tools/glycomod/).

### Localization of SRf1 by Immunostaining

Cyst tissues including protoscoleces were derived from infected cotton rats. Intestinal epithelial tissue harboring adult worms was derived from the small intestines of infected dogs on day 23 postinfection. The dogs were experimentally infected by oral administration of 5×10^5^ *E. multilocularis* protoscoleces, as mentioned above. The small intestine was divided into 6 sections, and the central sections of the intestine were cut out. All tissues were fixed in 10% formalin-PBS and embedded in paraffin wax. Cryosections (thickness, 4 µm) were cut on a Retoratome REM-710 microtome (Yamato Kohki Co., Ltd., Japan) at 25°C and mounted on slides.

The slides were rehydrated in Tris-buffered saline (TBS), and endogenous peroxidase was inactivated by 10 min of incubation in 0.3% hydrogen peroxide (H_2_O_2_). Samples were washed with TBS for 5 min and incubated with mouse anti-SRf1 antibodies at a dilution of 1∶3000 in 3% BSA/PBS for 1 h. After an additional washing step as above, the slices were incubated with EnVision (Dako Japan inc.) for 30 min. Slices were washed 3 more times, and the bound antibody was detected with diaminobenzidine (DAB) for 5 min. The stained samples were further stained with hematoxylin and washed with distilled water.

### Statistics

A generalized linear model (GLM) was used to model the differences between treatments in the vaccine trial using the ‘MASS’ package [26] in R version 2.15.2 (R Foundation for Statistical Computing, Vienna, Austria). Since the data were overdispersed (the mean parasite burden of each group was much less than the variance), a negative binomial distribution was applied. The optimal statistical model was chosen based on the lowest value for Akaike’s information criterion (AIC).

## Results

### 2D-PAGE and Western Blot Analysis

**Figure 1:**
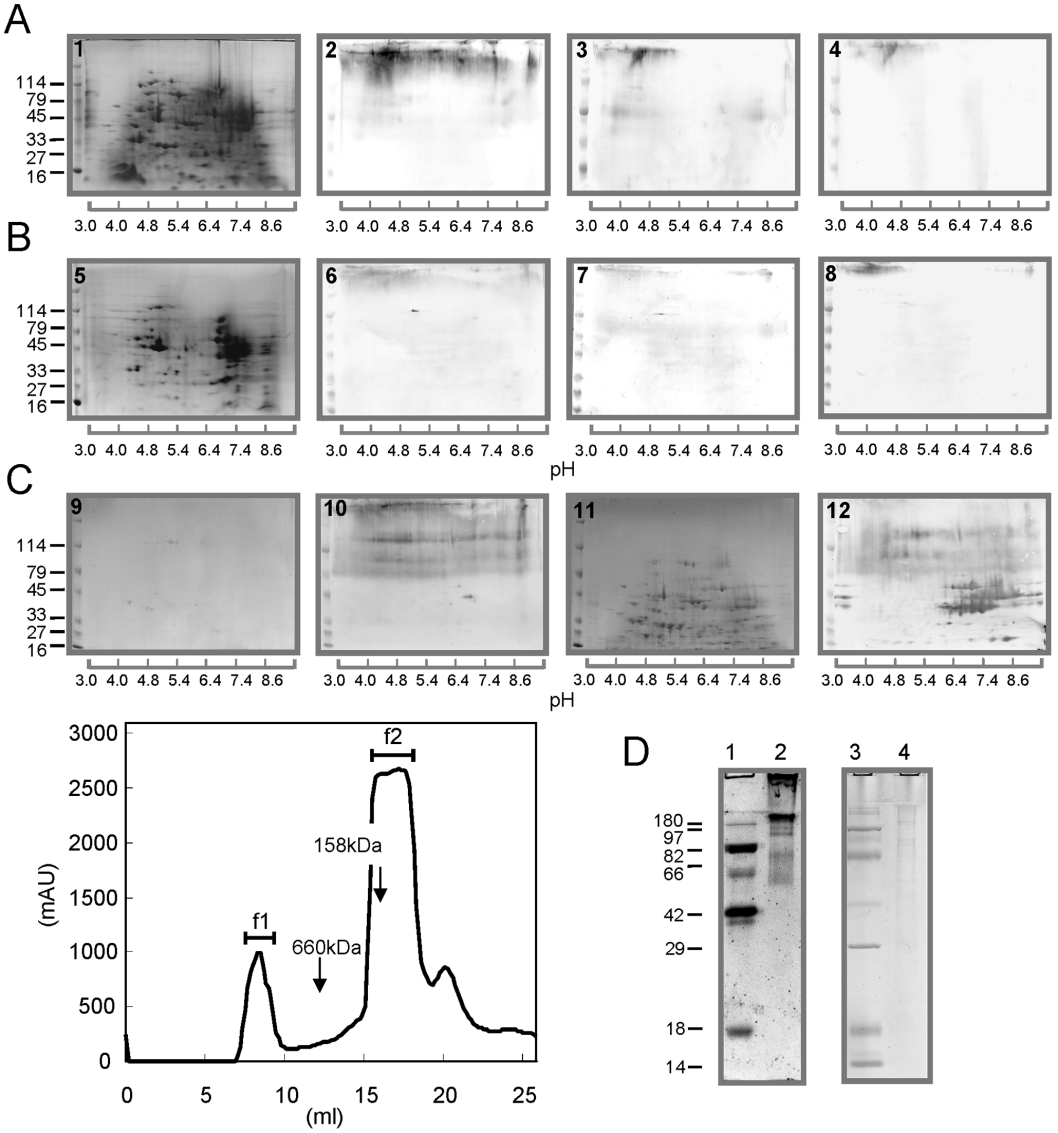
Identification and purification of vaccine antigen (SRf1) from *E. multilocularis*. Crude extracts were prepared from protoscoleces (PCE) and adult worms (ACE). A total of 120 µg protein was applied to 2D-PAGE. The proteins were blotted onto a PVDF membrane, and reactivity between proteins and intestinal IgA from dogs experimentally infected with *E. multilocularis* was examined. Panels A and B: PCE and ACE, respectively; panels 1 and 5: CBB-stained gels; panels 2 and 6: tested with intestinal IgA from dogs infected 5 times; panels 3 and 7: tested with intestinal IgA from dogs infected 3 times; and panels 4 and 8: tested with intestinal IgA from dogs infected once. Molecular size markers are indicated on the left (in kDa). Panel C: a gel filtration chromatogram of the vaccine antigen (SRf1) and 2D-western blot analysis; panels 9 and 11: CBB-stained gels of SRf1 and SRf2; panel 10: 2D-western blotting for SRf1 using intestinal swabs from dogs infected 5 times; panel 12: 2D-western blotting for SRf2 using sera from dogs infected 5 times. Panel D: SDS-PAGE analysis of SRf1; lanes 1 and 2: glycoprotein stained-gel of molecular size markers and SRf1; 3 and 4: CBB-stained gel with molecular size markers and SRf1. Glycoprotein detection was performed with a Pro-Q Emerald 300 gel stain kit and CandyCane glycoprotein molecular weight standards.

**Figure 2:**
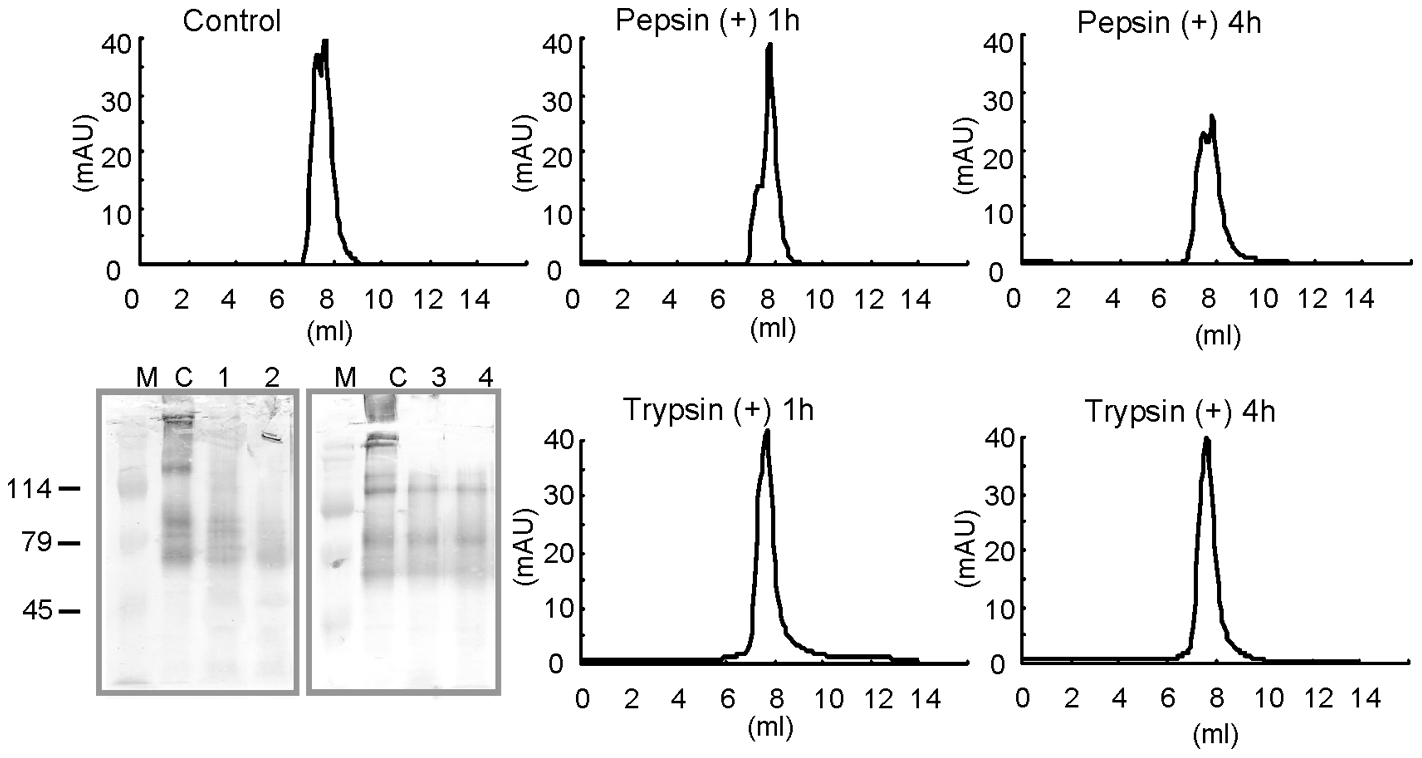
Tolerance of SRf1 against gastric protease digestion. SRf1 was digested in the presence of pepsin (1 mg/mL) at pH 2 or in the presence of trypsin (0.4 mg/mL) and chymotrypsin (1.7 mg/mL) at pH 7.4. After 1 or 4 h digestion, the reaction mixture was applied to a Superose 6 gel filtration column, and peak areas were compared to those of the control. Panel B: 1D-western blot analysis of digested SRf1s detected using sera from dogs infected 5 times. Lanes: M, molecular marker; C, controls (no proteases); 1 and 2, pepsin digestion for 1 and 4 h, respectively; 3 and 4, tryptic digestion for 1 and 4 h, respectively.

**Figure 3:**
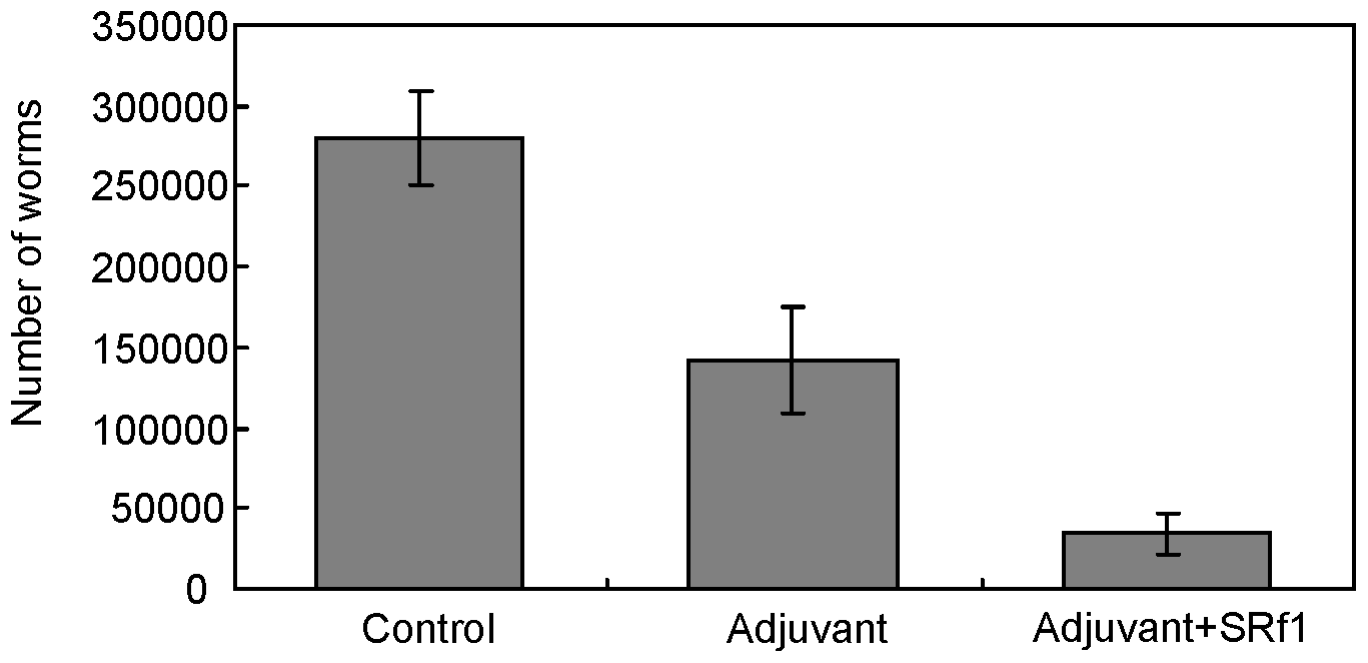
Challenge infection in dogs mucosally immunized with SRf1. Each dog was nasally immunized with SRf1 with CTB adjuvant 4 times. A booster was given orally 3 times with CT. Protoscoleces (5×10^5^) were administrated orally after the final immunization. As controls, groups were immunized with PBS or PBS plus adjuvant. The values are the mean number of adult worms ± the S.D. The model separating the group immunized with SRf1 plus adjuvant from the control group best fits the data according to generalized linear modeling (P<0.001).

**Figure 4:**
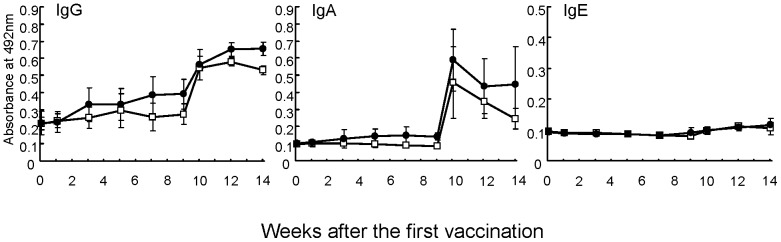
Serum antibody response in dogs immunized via the nasal and oral route with SRf1. Serum antibody responses against SRf1 evoked by nasal and oral immunization were detected by ELISA. The closed circle and open square indicate groups immunized with SRf1 plus adjuvant and adjuvant alone, respectively. Compared with the adjuvant control, an increase in the IgG response was detected in the group immunized with SRf1. No significant IgA or IgE responses were detected during immunization. A sharp increase in IgG and IgA responses were detected in each group after challenge infection.

**Figure 5:**
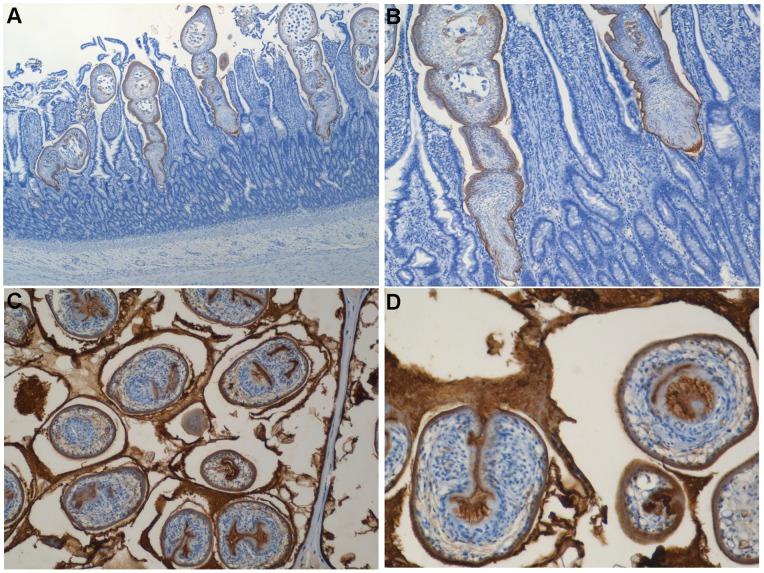
Localization of SRf1 in the larval and adult stages of *E. multilocularis*. Immunostaining was performed using polyclonal mouse anti-SRf1 antiserum. Panels A (40×) and B (100×) show adult worms harbored in the epithelium of the small intestine of infected dogs at 23 days postinfection. Panels C (100×) and D (200×) show protoscoleces in cysts derived from infected cotton rats. The brown color indicates specific antibody reactivity; the blue color indicates hematoxylin staining of nuclei. Anti-SRf1 antibodies were detected on the surface, including suckers, rostella, and hooks in both stages of worm development.

To search for vaccine candidates against *E. multilocularis* infection, reactivity of intestinal IgA from infected dogs to PCE or ACE was analyzed by 2D-PAGE and western blotting. Proteins from the PCE were separated on a 2D-gel with a pH gradient from 3 to 10. Approximately 250 protein spots were visualized by CBB staining (Fig. 1A–1). 2D-WB analysis demonstrated that all intestinal swab samples reacted with a smear band at the top of the membrane at a pI ranging from approximately 3.5 to 5 (Fig. 1A–2 to 1A–4), while no clear spots were detected with all intestinal swabs. These findings strongly suggest that intestinal IgA from infected dogs recognize glycoprotein(s) of the parasite. In addition, the intestinal swabs from dogs infected 3 or 5 times recognized a wider range of the smear band at the top of the membrane than the intestinal swab from dogs infected only once. Proteins from the ACE were separated on a 2D-gel as approximately 190 protein spots by CBB staining (Fig. 1B-5). All intestinal swabs from infected dogs reacted to a smear band from the ACE found at the same position as the reactive smear band from the PCE. The reactivity of all intestinal swabs to the ACE was significantly lower than their reactivity to the PCE, which suggested that the content of reactive antigens corresponding to the smear band in the PCE was higher than that of the ACE.

### Purification of the SRf1 Antigen

To isolate the reactive antigens corresponding to the smear band in Fig. 1A, the PCE was applied to a Superose 6 gel filtration column (Fig. 1C). Two major peaks were eluted at 7.5–9.5 mL for the first peak and 15–19.5 mL for the second peak. The eluted position of the first peak corresponded to the void volume of the column; therefore, the molecules in this peak were predicted to be over 4000 kDa, which was judged by the molecular size standard in an identical gel filtration chromatography assay. These peak fractions were collected and designated as fractions 1 (SRf1) and 2 (SRf2), respectively. 2D-PAGE analysis revealed that almost all nontarget proteins of the PCE were present in SRf2, while 5 weak protein spots were detected in SRf1 (Fig. 1C–9 and 11). The concentrations of these impurities were calculated to be 16.9, 11.0, 3.6, 3.6, and 1.2 µg/mL in SRf1 (1 mg/mL) by densitometric analysis (data not shown). Thus, the glycoprotein component (SRf1) was purified over 95%. 2D-WB analysis using intestinal swab samples from dogs infected 5 times revealed that SRf1 showed a similar pattern as that observed in Fig. 1A–2, indicating that SRf1 contained abundant amounts of target antigens (Fig. 1C–10). In contrast, serum samples collected from dogs infected 5 times showed significant reactivities to many proteins found in SRf2 (Fig. 1C–12).

In this experiment, the SRf1 components were not stained with CBB. Similar results have been reported for the mucin glycoprotein, which is highly glycosylated [27], [28]. In addition, the SRf1 components were always detectable by 2D-WB with infected dog sera [20] and glycoprotein staining with reagents from a Pro-Q Emerald 300 glycoprotein stain kit (Fig. 1D). Based on above-mentioned findings, we decided to use SRf1 for the vaccination experiments.

### Protease Tolerance of SRf1

To determine whether SRf1 retained its immunogenicity after gastric protease digestion, the tolerance of SRf1 to gastric protease digestion was examined by analytical gel filtration and 1-dimensional (1D) western blot analysis (Fig. 2). SRf1 was incubated with pepsin at 37°C for 1 or 4 h. The peak areas on gel filtration were calculated to be 28.8 and 26.6 mAU, respectively. The peak area of the control with no proteases was calculated to be 31.1 mAU. Approximately 80% of the SRf1 protein was still intact after a 4-h digestion in the presence of 1 mg/mL pepsin. SRf1 was also digested at 37°C in the presence of trypsin and chymotrypsin for 1 or 4 h. The eluted peak areas were calculated to be 31.2 and 30.0 mAU, respectively. Western blot analysis using serum samples from dogs infected 5 times revealed only slight degradation of SRf1 depending on the digestion time with proteases.

### Sequence Analysis of SRf1 *O*-glycans

Hülsmeier et al. reported that a major antigen of *E. multilocularis* was a mucin-type glycoprotein designated as Em2 [29]. However, information about carbohydrate moieties in the field of *E. multilocularis* research is still very limited. To address whether SRf1 was identical to the glycoprotein Em2, sequence analysis of SRf1 *O*-glycans was performed. MALDI-TOF MS spectra revealed that the carbohydrate moiety consisted predominantly of Hex and HexNAc, which were estimated with the GlycoMod Tool based on the mass spectra. Variations in the estimated carbohydrate moieties with S/N ratios over 4.0 are summarized in Table 1. Major peaks showing peak areas of over 1000 were Hex_2_, Hex_1_HexNAc_1_, Hex_2_HexNAc_1_, and Hex_1_HeNAc_2_. These estimated glycan compositions were consistent with the reported carbohydrate moieties of Em2. Peaks showing peak areas of over 500 corresponded to Hex_1_Pent_1_, HexNAc_2_, Hex_1_HexNAc_1_Sulph_1_, Hex_3_, Hex_2_NeuAc_1_, Hex_4_, Hex_3_HexNAc_1_, Hex_2_HexNAc_2_, and Hex_4_HexNAc_1_ and were not reported as Em2 carbohydrate moieties, with the exception of Hex_2_HexNAc_2_. A total of 28 peaks showing peak areas of over 100 in this assay were inconsistent with Em2 carbohydrate moieties. These results suggested that SRf1 shared some glycoprotein components with Em2, but was not identical in its glycoprotein composition.

**Table 1:** Estimated O-glycan compositions of SRf1 from mass spectrometric data.

Obsd. m/z	S/N	Area	δmass*	Estimated glycan composition
439.612	5.0	940	−0.54	(Hex)_1_(Pent)_1_
469.843	19.7	3095	−0.32	(Hex)_2_
510.9	9.3	1417	−0.29	(Hex)_1_(HexNAc)_1_
511.886	16.5	2578	0.696	(Hex)_1_(HexNAc)_1_
552.941	7.9	812	0.724	(HexNAc)_2_
572.96	4.3	383	0.765	(Hex)1(Pent)_2_
591.963	9.5	837	0.816	(Hex)_1_(HexNAc)_1_(Sulph)_1_
631.998	10.7	811	−0.22	(Hex)_3_
655.025	4.8	352	0.772	(HexNAc)_1_(NeuAc)_1_
673.039	71.3	5243	−0.2	(Hex)_2_(HexNAc)_1_
715.068	68.6	4513	0.798	(Hex)_1_(HexNAc)_2_
776.119	10	549	0.84	(Hex)_2_(NeuAc)_1_
794.115	12.5	634	−0.15	(Hex)_4_
818.143	6.2	301	1.837	(Hex)_1_(HexNAc)_1_(NeuAc)_1_
835.149	10.3	476	−0.15	(Hex)_3_(HexNAc)_1_
836.137	19.1	893	0.841	(Hex)_3_(HexNAc)_1_
877.165	14.1	569	0.843	(Hex)_2_(HexNAc)_2_
956.201	12	367	−0.12	(Hex)_5_
998.216	20.9	533	0.867	(Hex)_4_(HexNAc)_1_
1040.237	16.1	373	1.862	(Hex)_3_(HexNAc)_2_
1080.226	6.3	132	0.864	(Hex)_2_(HexNAc)_3_
1122.275	5.3	115	0.854	(Hex)_1_(HexNAc)_1_(NeuAc)_2_
1159.285	8.7	192	−0.07	(Hex)_2_(HexNAc)_3_(Sulph)_1_
1160.279	20.5	370	0.878	(Hex)_5_(HexNAc)_1_
1242.316	8.7	141	0.861	(Hex)_3_(HexNAc)_3_
1284.327	5.7	113	1.846	(Hex)_2_(HexNAc)_4_
1322.33	15.4	237	0.919	(Hex)_3_(HexNAc)_3_(Sulph)_1_
1404.365	9.8	166	0.858	(Hex)_4_(HexNAc)_3_
1446.377	7.1	129	1.843	(Hex)_3_(HexNAc)_4_
1556.409	9.7	163	1.817	(Hex)_2_(HexNAc)_2_(NeuAc)_2_(Sulph)_1_
1608.415	10.2	176	1.828	(Hex)_4_(HexNAc)_4_
1650.413	4.8	109	1.807	(Hex)_3_(HexNAc)_2_(NeuAc)_2_
1770.420	5	107	1.78	(Hex)_5_(HexNAc)_4_
1932.456	4.3	126	1.764	(Hex)_6_(HexNAc)_4_

* δmass = [observed m/z] – [theoretical m/z]. Abbreviations: Hex, hexose (e.g., mannose, galactose); HexNAc, N-acetylhexosamine (e.g., GlcNAc, GalNAc); NeuAc, N-acetylneuraminic acid; Sulph, sulfated glycan; Pent, pentose.

### Vaccine Trial of SRf1

The efficacy of SRf1 as a vaccine was evaluated by the reduction in the number of *E. multilocularis* adult worms in the small intestine of immunized dogs. Dogs were immunized nasally 4 times with CTB, and a CT booster was subsequently administered 3 times orally. After the final immunization, 5×10^5^ protoscoleces were administered orally to each group of dogs. The numbers of adult worms in challenge infection were follows: 298,675, 349,875, 169,875, 289,000, and 291,000 for the group immunized with PBS alone; 201,450, 215,850, 175,800, 41,800, 73,800, and 145,125 for the group immunized with adjuvant alone; and 210, 7,700, 37,675, 20,670, 64,550, and 77,000 the group immunized with adjuvant plus SRf1. Figure 3 shows the mean number of adult worms in each group. The control group immunized with the adjuvant alone showed a 49.1% reduction in the mean number of adult worms compared with that of the group immunized with PBS alone. The group of dogs immunized with adjuvant plus SRf1 developed fewer adult worms (corresponding to a 87.6% reduction) than the control group. GLM analysis indicated that the model separating the SRf1 plus adjuvant group from the other 2 groups (PBS and adjuvant only) was the best among the tested models, with the lowest AIC value of 433.76 (*P*<0.001). Thus, we provided the first direct experimental evidence that SRf1 induced a host protective response in *E. multilocularis* infection. No significant suppression of growth was observed in dogs immunized with adjuvant alone or in dogs immunized with adjuvant plus SRf1.

### Serum Antibody Response

Using serum samples collected during the course of immunization and infection, IgG, IgA, and IgE specific to SRf1 were examined by ELISA (Fig. 4). IgG levels in sera from dogs immunized with adjuvant plus SRf1 gradually increased from day 21 to day 63 after the first immunization, whereas no significant change was observed in sera from dogs immunized with adjuvant alone. On day 70, a sharp increase in IgG levels was observed in both groups, which corresponded to 7 days postinfection. Likewise, a sharp increase in IgA levels was observed in sera from dogs in both groups on day 70. No significant increase in IgA or IgE levels was observed in sera from dogs in both groups during the course of immunization. IgA could not be detected in saliva from dogs immunized with SRf1, but this was likely due to technical difficulties.

### Localization of SRf1

To determine the localization of SRf1 in *E. multilocularis*, immunostaining of adult worms and protoscoleces was performed using mouse antiserum against SRf1. Western blot analysis using the PCE or ACE revealed that mouse anti-SRf1 antiserum recognized SRf1 alone (data not shown). As shown in Fig. 5A and B, anti-SRf1 antibodies enabled the visualization of SRf1 on the surface of adult worms collected from the epithelium of the small intestines of infected dogs. Anti-SRf1 antibodies also stained the surface and apical region of protoscoleces, including suckers, rostella, and hooks (Fig. 5C and D). Thus, in both the adult and larval stage, SRf1 was considered to be expressed in the tegument, including the suckers, rostella, and hooks.

## Discussion

Despite the urgent need for new measures and tools to control *E. multilocularis* transmission, vaccine development for this parasite has been neglected [11]. The present study aimed to find a novel vaccine candidate that could induce protection against infection with adult *E. multilocularis* in definitive hosts. We found that a glycoprotein component, SRf1, purified from *E. multilocularis* protoscoleces by gel filtration, showed immunoreactivity with intestinal IgA in infected dogs. Moreover, mucosal immunization with this component induced significant reduction in worm burden in the immunized dogs. These findings suggested that further purification and immunological characterization of SRf1 could lead to the development of a novel vaccine candidate for the control of alveolar echinococcosis in humans.

In this study, we used combined nasal and oral mucosal immunization for delivery of the antigen. Some studies have investigated the immunoresponse in dogs immunized via mucosal administration of antigen candidates against *Echinococcus* infection. Carol et al. demonstrated that nasal immunization of immunostimulating complexes made from the *E. granulosus* tegumental antigen from protoscoleces showed significant induction of the secretory IgA antibody response detected in saliva and serum from dogs infected with *E. granulosus* [30]. Additionally, Gottstein et al. reported that subcutaneous and peroral vaccinated dogs showed strong humoral immune responses to antigens [31]. In both of these reports, no challenge infection data was available. However, Carol et al. suggested that more stringent and innovative search methods for appropriate immunogens or adequate immunization regimes were needed for successful development of protection against infection. Therefore, we decided to use a unique approach to identify vaccine candidates based on the reaction between parasite antigens and intestinal IgA from dogs repeatedly infected with *E. multilocularis*.

A series of egM recombinant antigens showed reactivity to the sera from dogs infected with *E. granulosus* [32]. EgA31 also possessed significant reactivity to the sera from infected dogs [33]. These facts indicated that these vaccine candidates, in their native forms in the infected parasite, were recognized by mucosal antigen-presenting cells in the small intestines of dogs infected with *E. granulosus* and induced a systemic antibody response. Such serum reactivity is important for vaccine candidates, and this was also observed in our study of SRf1. In our previous report, large glycoproteins with reactivity to sera from dogs infected with *E. multilocularis* were identified by the 2D-WB method [20]. Here, SRf1 was detected on the top of the membrane as a smear, at the same position as the large glycoproteins on 2D-WB analysis. Therefore, this suggested that SRf1 was recognized by both serum IgG and intestinal IgA from dogs infected with *E. multilocularis*. Similarly, Zhang and McManus [11] found local mucosal and systemic immune responses generated against *E. granulosus* in infected dogs. In addition, SRf1 showed significant tolerance against gastric proteases. Taken together, these data suggest that effective vaccine antigens, capable of oral administration, can be generated against *Echinococcus* infection.

In a preliminary experiment using 6 dogs, subcutaneous administration of SRf1 with Freund’s adjuvant did not show a significant level of protection, although the immunized dogs exhibited a significant IgG response in sera as well as obvious physiological responses, such as diarrhea and inflammation of the skin (data not shown). In this study, to identify an adequate immunization regime, we used mucosal immunization combined with 4 nasal and 3 oral immunization doses. As a result, administration of SRf1 with mucosal adjuvant induced an 87.6% reduction in worm numbers in the immunized group compared to the control group, indicating that SRf1 was a promising antigen for use as a mucosal vaccine against *E. multilocularis* in dogs. In addition, to prevent transmission of *E. multilocularis*, the development of a deliverable oral vaccine, such as in fox baits, like the rabies vaccine, is required. Therefore, studies are needed to further support the immunogenicity of SRf1 and its application as a bait oral vaccine.

In this study, we also examined the effectiveness of SRf1 by using CTB and CT, some of the strongest mucosal adjuvants available. Many researchers have attempted to develop mucosal vaccines using nasal or oral administration of antigen with CTB or CT as a mucosal adjuvant [34], [35]. Pierce et al. evaluated CT as an oral immunogen against experimental canine cholera. Dogs were immunized orally with a 0.1 mg dose of purified CT and demonstrated marked protection. However, they reported that most dogs experienced moderate diarrhea following administration of CT [35]. Unexpectedly, no symptoms were observed in all experimental dogs in our study. This may be due to differences in the route of administration for CT. Unfortunately, the use of CT is limited by its promiscuous binding to GM1 ganglioside receptors present on all nucleated cells, including epithelial cells and nerve cells [36]. Indeed, a commercial intranasal flu vaccine with a CT as an adjuvant revealed an increased incidence of Bell’s palsy in vaccinated subjects [36]. An alternative strategy has proven that mutant CT, which has no or very little enzyme activity, can act as a mucosal adjuvant. CpG oligonucleotides [37] would be also a better strategy for mucosal vaccine development.

In this study, a weak but clear IgG response specific to SRf1 was observed in the immunized group (adjuvant plus SRf1), whereas no specific antibody response was observed in the control group. Mucosal immunization with antigen, co-administered with a mucosally active adjuvant, such as CT, induces both systemic and mucosal immunity [36]. In this study, the mucosal IgA response evoked by immunization of SRf1 could not be detected, most likely due to technical difficulties. However, consistent with a previous study [38], we observed a sharp increase in serum IgG and IgA responses just after challenge infection. Moreover, Tanaka et al. observed a similar antibody (IgG and IgA) response in sera from dogs experimentally infected with *E. multilocularis*. However, the detailed mechanisms through which this process leads to protection are still unknown.

Immunostaining revealed that the SRf1 antigen localized at the surface of both larval and adult forms of *E. multilocularis* (Fig. 5). Notably, SRf1 was also detected on the suckers and rostella, suggesting that SRf1 was recognized by the intestinal immune system during the course of infection. In a previous study, the EgA31 clone was shown to encode a paramyosin protein that also showed very similar localization in adult *E. granulosus* organisms [33]. Thus, surface antigens could represent a class of strong immunogens that are able to induce protection via the mucosal immune response. In addition, a significant reduction in worm numbers was observed in the adjuvant-immunized group. In line with this, infection with the gastrointestinal nematode *Strongyloides* is known to induce host protective immunity by accumulation of mucosal mast cells and activation of mucin release from goblet cells [39], [40]. Studies have also suggested that mucin release is induced by CT via interactions with intestinal goblet cells [41], [42]. This nonspecific protection induced by mucosal immunization with CTB and CT may provide insights to promote our understanding of the mechanisms of protection induced by mucosal immunization.

Our glycosylation analysis and immunostaining results revealed that the SRf1 antigen could be distinguished from Em2, a known mucin-type glycoprotein that localizes to the laminated layer of metacestode-stage *E. multilocularis* worms. The SRf1 antigen comprised 1 or more highly glycosylated tegument proteins.

Here, we provide evidence that SRf1, a large glycoprotein component from *E. multilocularis* protoscoleces, has vaccine potential to induce significant reduction in the worm burden in experimentally immunized dogs. However, this component, obtained by a relatively simple procedure, does not consist of a single molecular structure, as shown in our results. Thus, further purification and immunological characterization should be performed to identify the precise molecular component that is responsible for inducing the protection. Additionally, this vaccine candidate should be examined for its dose dependency and longevity of efficacy after immunization by performing experimental challenge infections. Successful characterization of the molecular structure of the vaccine candidate would open the way to large-scale preparation of the material by *in vitro* expression or synthesis, which is essential not only for further experimental studies but also for practical application in controlling the parasite.
